# Chemical exposures from upholstered furniture with various flame retardant technologies

**DOI:** 10.1111/ina.12805

**Published:** 2021-02-24

**Authors:** Aika Davis, P. Barry Ryan, Jordan A. Cohen, Debra Harris, Marilyn Black

**Affiliations:** ^1^ Underwriters Laboratories, Inc. Marietta GA USA; ^2^ Emory University Atlanta GA USA; ^3^ Baylor University Waco TX USA

**Keywords:** dermal, exposure, flame retardants, ingestion, inhalation, upholstered furniture, volatile organic compounds

## Abstract

Upholstered furniture is often manufactured with polyurethane foam (PUF) containing flame retardants (FRs) to prevent the risk of a fire and/or to meet flammability regulations, however, exposure to certain FRs and other chemicals have been linked to adverse health effects. This study developed a new methodology for evaluating volatile organic compound (VOC) and FR exposures to users of upholstered furniture by simulating use of a chair in a controlled exposure chamber and assessing the health significance of measured chemical exposure. Chairs with different fire‐resistant technologies were evaluated for VOC and FR exposures via inhalation, ingestion, and dermal contact exposure routes. Data show that VOC exposure levels are lower than threshold levels defined by the US and global indoor air criteria. Brominated FRs were not detected from the studied chairs. The organophosphate FRs added to PUF were released into the surrounding air (0.4 ng/m^3^) and as dust (16 ng/m^2^). Exposure modeling showed that adults are exposed to FRs released from upholstered furniture mostly by dermal contact and children are exposed via dermal and ingestion exposure. Children are most susceptible to FR exposure/dose (2 times higher average daily dose than adults) due to their frequent hand to mouth contact.

**Practical Implications**

- A method was introduced for evaluating potential human risks associated with flame retardants and/or other chemical exposures from a single household product, in this case study with upholstered furniture. Each household product is isolated inside an environmentally controlled exposure chamber and operated to simulate normal use as chemical emission rates are released and measured.
- The method aids in identifying sources and routes of human exposure to flame retardants and other chemicals of concern that may be associated with household products. Quantified emission rates contribute to finding the relationship between emission sources and empirical concentrations measured in indoor environments and human matrices (eg, serum, urine, breast milk, tissue, hair, nail).
- Upholstered furniture can be protected from fire hazards and chemical pollutant exposure without compromising utility using currently available technologies, for example, use of barrier textile between the cover fabric and foam of a chair.

## 1 INTRODUCTION

Flame retardants (FRs) have been added to consumer products since the 1970s to reduce the risk of residential fires. The use of FRs has evolved since then in the United States (US) due to key events. These included the 2004 phase out of commercial pentabromodiphenyl ether (pentaBDE) and octabromodiphenyl ether (octaBDE), and the 2010 voluntary phase‐out that additionally included decabromodiphenyl ether (decaBDE) and congeners of penta and octaBDEs after research showed that polybrominated diphenyl ethers (PBDEs) were persistent, bioaccumulative, and toxic.^1^ The phase‐out shifted the market to use alternative FRs such as the chlorinated FRs and eventually to organophosphorus FRs (OPFRs), shifting as research found adverse health impacts with other halogenated FRs.^2^ Currently, the use of OPFRs is on the rise, while their toxicological hazard has yet to be well‐characterized.^2^ Studies have demonstrated that some OPFRs affect the endocrine system, behavioral development and preterm birth, respiratory outcomes like asthma, and allergic disease.^3^ OPFRs can leach out of products over time and accumulate in settled dust in the surrounding environment.^4^, ^5^, ^6^ Humans are exposed to OPFRs as they are found on human hands^7^ and shown to metabolize in the human body.^8^, ^9^ In addition to the use of OPFRs as FRs, these chemicals are used in plasticizers, paints, glues, and for industrial processes. These organophosphates are prevalent and persistent in both indoor and outdoor environments.^10^

In addition, volatile organic compounds (VOCs) are ubiquitous, and consumer products emit numerous VOCs. For this reason, indoor air has a complex mixture of VOCs many with higher concentrations than outdoor air.^11^ Potential health impacts vary for specific VOCs based on exposure including cancer, reproductive harm, sensory irritation, odor annoyance, and headache.^12^, ^13^ Individuals spend 80% or greater of their day indoors and at least 66% of that in their residence.^14^ This results in spending the majority of time in environments contributing to VOC and potential FR exposures.

Flammability standards for furniture, when they exist, vary by country. There is no national flammability standard in the US, but England, for example, has an open flame flammability standard.^15^ The risk of fire continues to exist as domestic fires account for a large percentage of civilian fire deaths in both England^16^ and the US.^17^ For example, home fires caused 2720 civilian fire deaths, or 74% of the total fire deaths, in the US in 2018.^17^ The furniture flammability standard in California shifted from a required open flame performance test to a smolder resistance test^18^ which allowed products to be constructed and sold to the marketplace with little to no added FRs.

Studies coupling chemical exposure and fire performance risks of upholstered furniture manufactured with and without FRs are limited. This study investigated how humans may be exposed to VOCs and FRs from upholstered furniture manufactured with different FR technologies that currently exist in the market. This paper focuses on the exposure assessment component of a larger study on furniture flammability and human exposure to FRs, where chemical emissions into the surrounding environment during typical use of a lounge chair were examined using an exposure chamber and measuring inhalation, ingestion, and dermal contact exposures. The second portion of the study investigated flammability performance of the differently constructed lounge chairs and the relationship between daily exposure to FRs and fire hazards.^19^

## 2 METHODS

The research protocol consisted of three parts: design and acquisition of upholstered lounge chairs; simulated inhalation, ingestion, and dermal contact exposure measurements; and human exposure analysis and assessment.

### 2.1 Upholstered furniture

**FIGURE 1:**
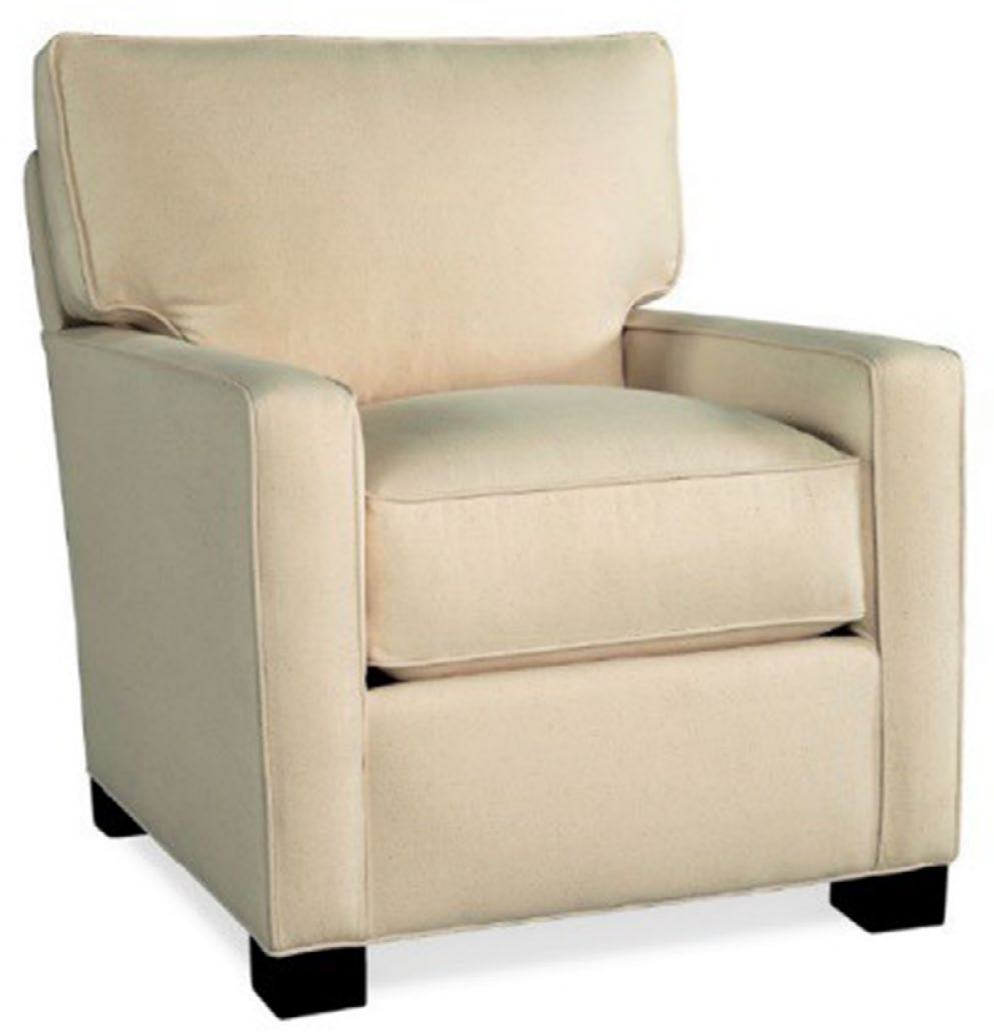
Upholstered chair design

Upholstered furniture used in this study (Figure 1) was a single person upholstered lounge chair, locally constructed for this study. The model of the chair is commercially available, and study chair construction followed the manufacturer's typical fabrication methods and materials (Table 1).

**TABLE 1:** Upholstered chair components

Components	Material
Cover textile	Cotton
Ticking textile	Cotton + PET
Fiber filling	PET
Foam (varies by FR technologies)	Polyurethane with or without FR
Poly loose filling	PET
Fiberglass barrier textile (only for barrier chair)	Woven glass fiber
Decking textile	Cotton +PET
Framing	Wood +metal

Abbreviation: PET, polyethylene terephthalate.

Chairs used in this study varied only by the differing FR technologies. The four FR technologies used were as follows:

1. NFR‐ No FR added to the polyurethane foam (PUF)/control
2. OPFR‐ A commercial FR added to the PUF, identified as *tert*‐butylated triarylphosphate esters (TBPP) mix
3. RFR‐ A proprietary reactive (polymer integrated) chemical FR used in forming the PUF
4. BNFR‐ No FR added to the PUF, but a barrier fabric material with no FR added between the PUF and textile cover

The details of the chair construction are in Supporting Information 1. In summary, the TBPP mix was identified to be 58% by weight of (4‐tert‐butylphenyl) diphenyl phosphate (4tBPDPP), along with 30% by weight of TPHP, 11% by weight of (2,4‐di‐tert‐butylphenyl) diphenyl phosphate (B4tBPPP), and 1% by weight of tris (4‐tert‐butylphenyl) phosphate (T4tBPP). The OPFR foam was found to contain 2.9% by weight of the TBPP mix. The RFR technology was expected to reduce leaching or migration of the FR from the product into the environment. The BNFR chair was fabricated without FRs but the PUF was wrapped and sewn with a woven fiberglass textile barrier which was placed directly under the cover textile.

### 2.2 Exposure methods

All newly manufactured chairs were tested for VOC and FR chemical emissions inside a dynamic, environmentally controlled exposure chamber. Air, dust, and simulated skin transfer samples were collected during simulated chair use and analyzed for exposure levels.

#### 2.2.1 Exposure chamber

**FIGURE 2:**
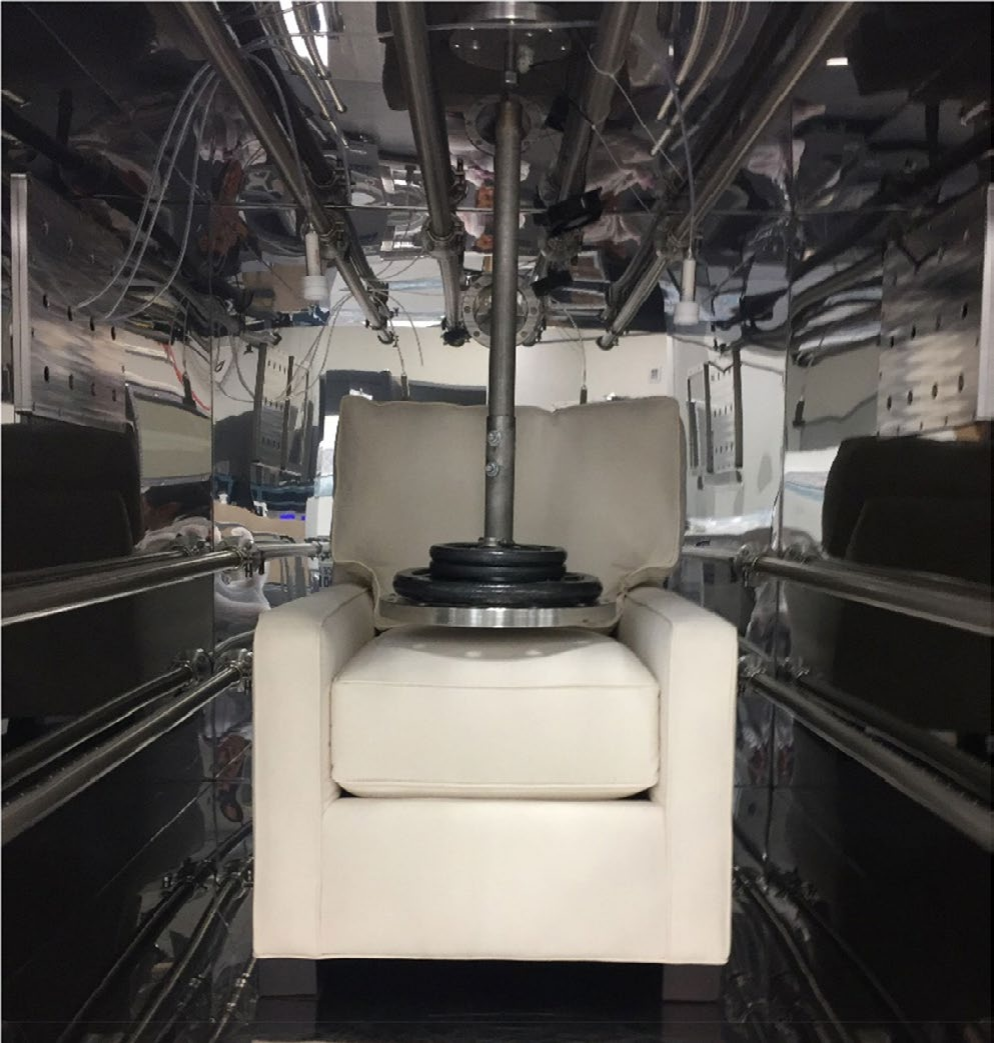
Chair and the automated sitting mechanism in an exposure chamber

Each chair was tested in a 6 m^3^ specialized exposure chamber (Figure 2) with dynamic air flow and controlled environmental conditions. The chamber operated as a single‐pass system without recirculation of chamber air and created an airtight seal with a closure mechanism with gaskets. All materials inside the chamber and chamber parts are made with low‐emitting products; the chamber walls and air flow system components were constructed with electropolished stainless steel. The clean supplied air concentration remained below 10 μg/m^3^ of total VOC (TVOC), 2 μg/m^3^ of any individual VOC, 1 μg/m^3^ of total particle mass concentration, and 2000/cm^3^ of total particle count concentration. The air exchange rate was operated at 1 ± 0.05 air change per hour (ACH), maintaining temperature at 23 ± 1°C and relative humidity at 50% ± 5%. An automated pneumatic sitting mechanism (Figure 2) simulated sitting activity during personal use of the chair. A 56.7 kg weight was dropped once every minute from 3.6 cm above the seat cushion to mimic an average US male's upper body sinking into the chair from a standing position. Chamber operation and the sitting device are based on existing standards mentioned in Supporting Information 2.

#### 2.2.2 Exposure testing

**FIGURE 3:**
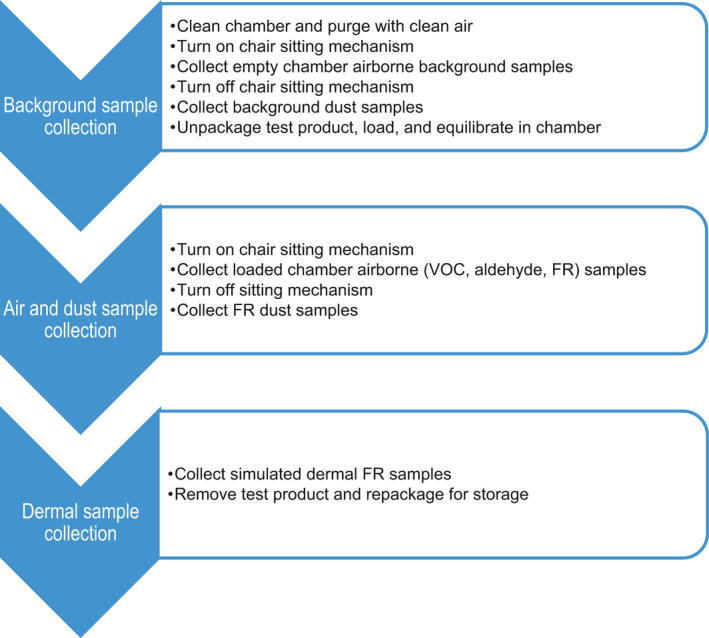
Exposure chamber sampling timeline

A typical exposure simulation and sample collection timeline is shown in Figure 3. Each test product required a total of 4 days to measure emissions of VOCs in air and FRs in air, settled dust, and simulated dermal contact. The first phase of sampling was background contaminant collections. The chair sitting mechanism was turned on in the empty chamber during the background sample collections. Air sampling was conducted for VOCs, aldehydes, and FRs in gas and particle phases. Immediately after the background airborne samples were collected, the chamber door was opened and the background dust wipe samples were collected. For the second phase of testing, the upholstered chair was introduced inside the chamber and equilibrated overnight before the sampling began. Then the sitting mechanism was turned on simulating the behavior of sitting activity on the chair for 24 h as VOCs and airborne FR samples were collected, followed by the collection of dust wipe samples. The simulated dermal FR transfer/skin absorption samples were collected from the chair seat cushion immediately after completion of air and dust sample collections. The interior surface of the chamber was wiped with deionized water and purged overnight with clean air to prepare for the next background sampling.

##### 2.2.2.1 Air exposure

**FIGURE 4:**
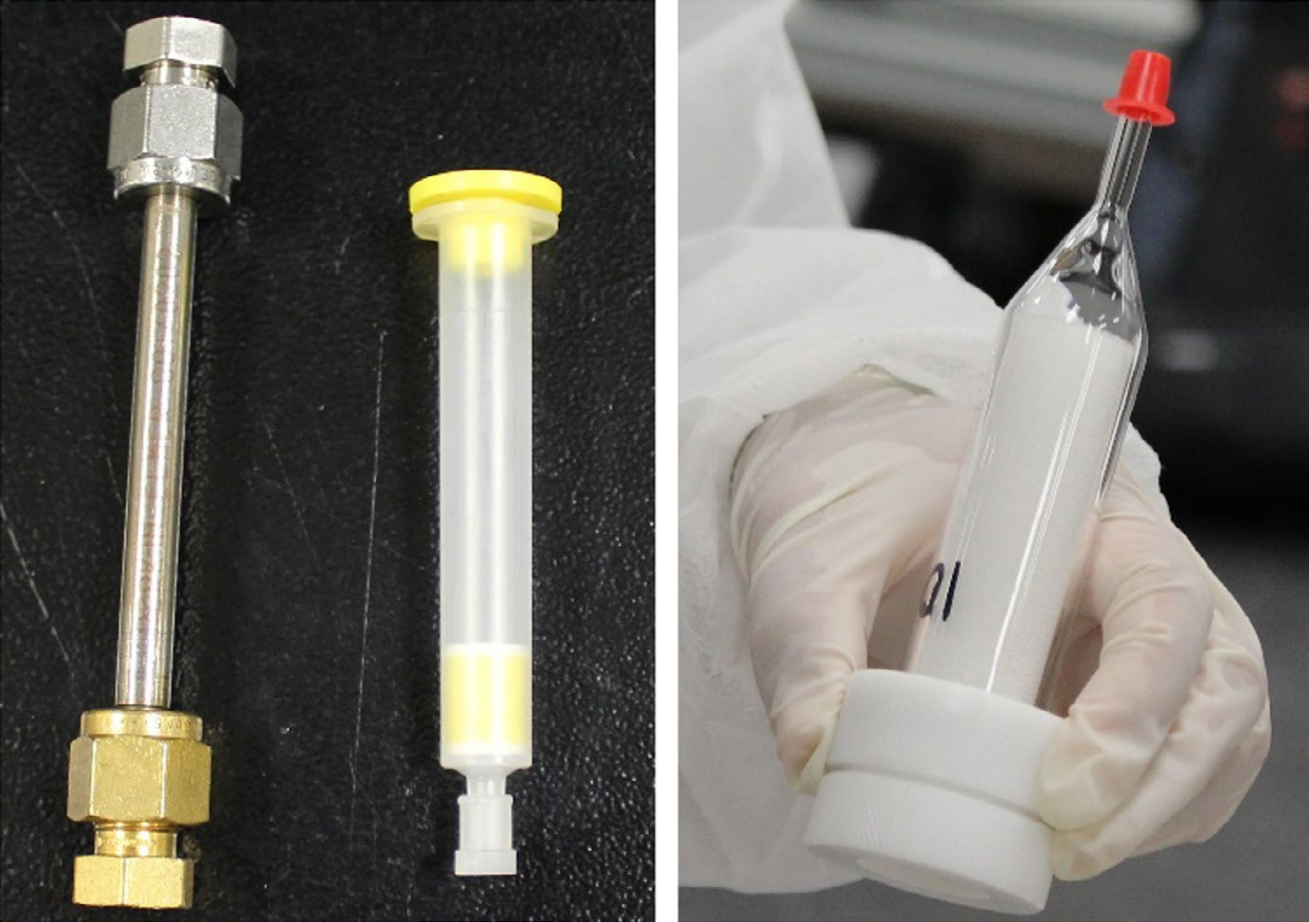
Environmental exposure sampling media for air sampling: Tenax® tube (left) and 2,4‐dinitrophenylhydrazine (DNPH) cartridge (middle) for volatile organic compounds (VOCs) and aldehydes respectively, and quartz filter in line with PUF cartridge for airborne FR (right)

Volatile organic compound samples were collected directly from inside the chamber onto Tenax® solid sorbent cartridges (Figure 4) using a system of pumps with in‐line mass flow controllers. VOC samples were collected for 90 min at 0.2 L/min (18 L collection volume) during background and at the beginning and end of the 24‐h FR airborne sampling period. Sampled sorbent tubes were thermally desorbed and analyzed by GC/MS, a method applicable for C_5_ to C_17_ organic chemicals with boiling points ranging from 35°C to 250°C.

Emissions of low molecular weight aldehydes up to hexanal were collected onto solid sorbent cartridges treated with 2, 4‐dinitrophenylhydrazine (DNPH) for 90 min at 0.5 L/min (collection volume of 45 L). Analysis was by high‐performance liquid chromatography (HPLC). 10% duplicates were collected for VOC and aldehyde samples. The detection limit for most VOCs and aldehydes was less than or equal to 0.5 μg/m^3^ and the limit of quantification (LOQ) was at or lower than 2 μg/m^3^, therefore a LOQ value of 2 μg/m^3^ was applied for all reportable chemicals. Additional details on GC/MS and HPLC analyses are in Supporting Information 2.

Airborne FR samples were collected for 24 h using a sampling train consisting of a quartz fiber filter designed to capture particulate phase (>2.5 µm in aerodynamic diameter) semi‐volatile FRs, followed by a manufacturer‐precleaned PUF cartridge capturing semi‐volatile FRs in airborne and particulates smaller than 2.5 µm in aerodynamic diameter (Figure 4) in line with an air sampling pump. A target air collection volume was 5760 L, and each sample was collected in duplicate. This sampling method was based on EPA Indoor Exposure Product Testing Protocols,^20^ and EPA Method TO‐10A.^21^

The FR chemical extraction method was based on protocols developed by van der Veen et al.^22^ In this method, two separate extractions were utilized to assess the different FRs of interest. Once the two fractions were eluted by different solvents (*n*‐hexane for PBDE congeners and ethyl acetate for OPFRs), the samples were evaporated dryness and reconstituted into a single solution in order to improve analyte throughput. The combined solution was analyzed using GC followed by electron impact ionization and MS. This extraction/analysis method was applied for settled dust and simulated dermal exposure samples as well. LOQ was set to one standard deviation of the blanks. List of target analytes as well as details on extraction, analysis, and QA/QC of FRs are in Supporting Information 3.

##### 2.2.2.2 Dust exposure

**FIGURE 5:**
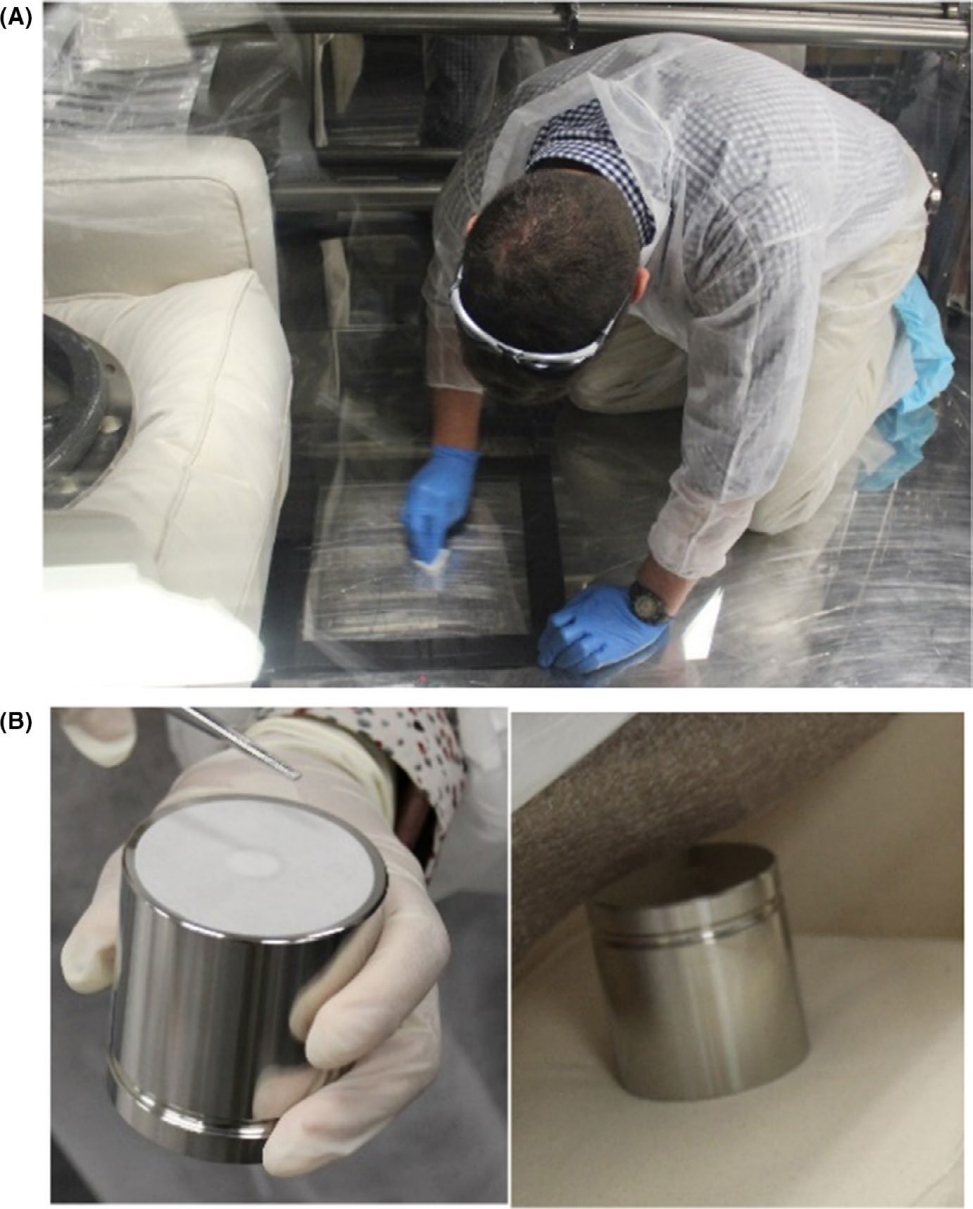
(A) Settled dust collection from the chair for FR analysis, and (B) simulated dermal transfer sample collection from chair with the filter patch (left) and filter placed between seat cushion and weight (right)

Settled floor dust around the test sample was collected using a wipe sample immediately after opening the chamber door after 24 h of chair agitation by the sitting mechanism, and the FRs in the dust sample were analyzed using the same method as for airborne FRs. A fixed surface area was sampled using a defined 0.093 m^2^ template and sterile gauze impregnated with n‐hexane (Figure 5a). The same surface area was wiped three times in three different directions. Settled dust samples were collected in duplicate; one wipe sample was collected in front of the chair and the other to the side of the chair. The sampling method is based on EPA‐740‐R‐13‐001.^23^ Since the sitting mechanism mechanically mimicked one sitting per minute inside the chamber, this equates to about 3 months of chair use through agitation prior to collecting dust samples.

##### 2.2.2.3 Dermal exposure

The simulated dermal sampling method was developed based on EPA Indoor Exposure Product Testing Protocols,^20^ Thomas et al,^24^ and EPA Guideline 875.2300.^25^ Dermal transfer of FRs from the test product surface was simulated using a patch protocol, and FRs in the sample were analyzed as stated above. A filter paper patch impregnated with a 0.9% saline solution was placed on the seat of the test chair (Figure 5b). A stationary weight was placed on top of the patch for 6 h to mimic an average person sitting on top of a specific surface area.

### 2.3 Exposure prediction

Expected human exposure levels and doses of FRs through oral, dermal, and inhalation routes were calculated using defined exposure models. The mathematical exposure models were based on the approach of Keil et al,^26^ and the model parameters were obtained from the EPA Exposure Factors Handbook^14^ and other cited literature. Exposure modeling was performed for three personal physiological models: adult, toddler (1–2 years old), and infant (3–6 months old). The equations and parameters used for calculating average daily doses (ADDs) via inhalation, ingestion, and dermal contact exposure predictions are presented in Supporting Information 4.

## 3 RESULTS

### 3.1 VOCs

Measured TVOC emission rates (ERs) among the chairs ranged from 310 to 1090 μg/h and 44 individual VOCs were identified above the LOQs (Table 2, full list in Supporting Information 5 Table S6). Similar compounds were emitted from the four types of chairs including hexanal (VOC of highest emission rate), propanoic acid, pentanal, hexanoic acid, acetaldehyde, 2‐butoxyethanol, 2‐ethyl‐1‐hexanol, and formaldehyde. Sources of these included the construction materials, textiles, PUF, wood frame, and adhesives. The flammability construction technologies had little impact on the individual VOC emissions. PUF related VOC were measured including propylene carbonate, tetradecamethylhexasiloxane, and 1‐[2‐(2‐methoxy‐1‐methylethoxy)‐1‐methylethoxy]‐2‐propanol. VOC analytical measurement duplicates were within a 20% difference.

**TABLE 2:** TVOC and top ten individual VOCs and their average emission rate (μg/h/chair)

CAS No	Chemical	No FR (control)	OPFR	Reactive FR (RFR)	Barrier textile (BNFR)
Total	TVOC	596	1090	310	794
66‐25‐1	Hexanal	322	563	116	428
79‐09‐4	Propanoic acid	76.1	194	145	191
7136‐3	1‐Butanol	170	24.9	22.2	230
108‐32‐7	Propylene Carbonate	NQ	284	NQ	NQ
110‐62‐3	Pentanal	57.4	114	18.0	93.0
123‐38‐6	Propanal	43.7	117	NQ	68.7
142‐62‐1	Hexanoic acid	32.3	33.9	20.6	69.3
108‐95‐2	Phenol	NQ	179	NQ	NQ
75‐07‐0	Acetaldehyde	62.9	50.4	17.7	66.3
111‐76‐2	Ethanol, 2‐butoxy	45.5	44.4	28.5	68.7

Abbreviation: NQ, not quantifiable since chamber concentrations used to calculate the emission rate were below LOQ of 2 μg/m^3^.

The predicted VOC exposure concentrations were calculated from the highest ERs in Table 2 for each chemical as a conservative scan for risk. The predicted exposure concentrations in a scenario with the living/dining room with an air exchange rate of 0.45/h and another in a small bedroom with low ventilation, which represented the worst case in a residential setting, are presented in Table 3 (Supporting Information 5 Table S7 for a full list). Maximum predicted living/dining room concentrations were calculated using the maximum emission rates across all chairs with 201 m^3^ space^27^ with 0.45/h air exchange rate (average for US residential homes),^14^ one chair per room. Maximum predicted bedroom concentrations were calculated using the maximum emission rates across all chairs with 28.2 m^3^ space^27^ with 0.23/h air exchange rate (for a high performance/energy efficient home).^28^ The predicted concentrations were compared with key globally accepted criteria for indoor air quality (Table 3).

**TABLE 3:** TVOC and top ten predicted individual VOC concentrations (in a living room and a bedroom) and criteria by Ausschuss zur gesundheitlichen Bewertung von Bauprodukten's Lowest Concentration of Interest (AgBB LCI),^29^ the American Conference of Governmental Industrial Hygienists' Threshold Limit Values (ACGIH TLV®),^33^ and the California Department of Public Health Standard Method (CDPH SM).^32^ All concentrations are in μg/m^3^

CAS No.	Chemical	Living room conc.	Bedroom conc.	AgBB LCI	ACGIH 1/10 TLV	CDPH SM
000‐00‐0	TVOC	12.1	168	NA	NA	NA
66‐25‐1	Hexanal	6.22	86.8	900	NA	NA
79‐09‐4	Propanoic acid	3.34	46.6	310	NA	NA
71‐36‐3	1‐Butanol	2.54	35.5	3000	6060	NA
108‐32‐7	Propylene carbonate	3.14	43.8	250	NA	NA
110‐62‐3	Pentanal	1.26	17.6	800	NA	NA
123‐38‐6	Propanal	1.29	18.0	NA	NA	NA
142‐62‐1	Hexanoic acid	0.80	11.2	490	NA	NA
108‐95‐2	Phenol	1.98	27.6	10	1930	100
75‐07‐0	Acetaldehyde	0.73	10.2	1200	4500	70
111‐76‐2	Ethanol, 2‐butoxy	0.76	10.6	1100	9670	NA

Abbreviation: NA, not available.

Thirty‐one VOCs known as carcinogens, irritants, reproductive or developmental toxins^29^ were measured from the four types of chairs. Acetaldehyde, a suspected carcinogen,^30^, ^31^ and formaldehyde, a class 1 carcinogen,^30^ were emitted from all chair types. The predicted concentrations for acetaldehyde in both the living room (0.7 μg/m^3^) and the bedroom (10.2 μg/m^3^) were lower than the lowest guidance level of 70 μg/m^3^ from the California Department of Public Health Standard Method (CDPH SM)^32^ with one chair in the room. The predicted formaldehyde concentrations in the living room were one‐tenth of the lowest guidance level (9 μg/m^3^ from CDPH SM) regardless of air exchange rates, however formaldehyde concentrations in the bedroom (3.07 and 6.01 μg/m^3^ for average and high performance/lower ventilation respectively) were approaching the limit with one chair. Two chairs in the bedroom with low ventilation was predicted to exceed both CDPH SM limit and one‐tenth of the American Conference of Governmental Industrial Hygienists' Threshold Limit Values (ACGIH TLV®). An estimated safety factor of one‐tenth is applied to the ACGIH TLV values for differences between occupational and residential settings. Three chairs in the average ventilation condition would exceed the formaldehyde CDPH SM limit. Hexanal, propanoic acid, 2‐butoxyethanol and 2‐ethyl‐1‐hexanol are irritants that were consistently detected in all four types of chairs; however, their predicted exposure concentrations were lower than the guidance levels.

### 3.2 FR

**FIGURE 6:**
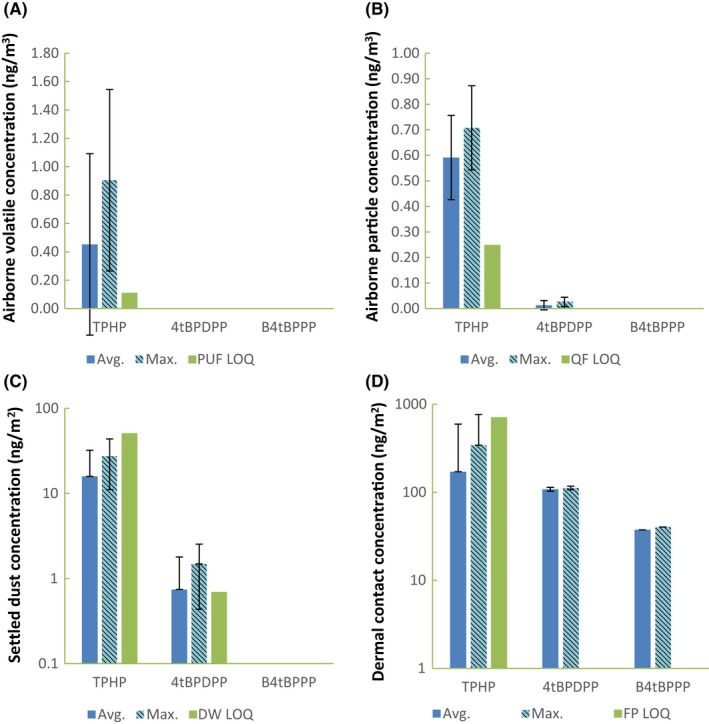
FR concentrations measured in the exposure chamber from OPFR chairs. The average of duplicate samples (with standard deviation) and the maximum FR concentrations for (A) inhalation route via semi‐volatile phase in PUF sampler (PUF), (B) inhalation via aerosol phase in quartz filter (QF), (C) ingestion rote sampled by dust wipe (DW) on the floor of the chamber (y‐axis in log scale), and (D) simulated dermal contact sampled via filter patch (FP; y‐axis in log scale) are presented along with LOQs. LOQ was set at three times LOD for those not clearly visible

**FIGURE 7:**
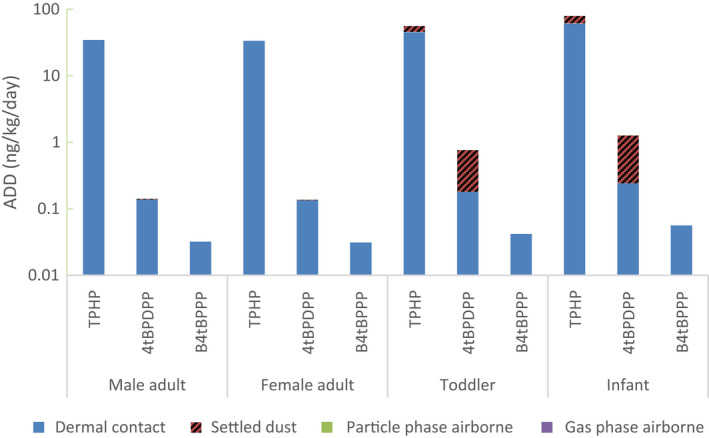
Predicted average daily doses (ADDs) in ng/kg/day for male, female adults, toddler, and infant. ADDs are separated by gas phase and particle phase inhalation, ingestion, and simulated dermal exposure routes. TPhP: triphenyl phosphate (using ABS of 0.18), 4tBPDPP: (4‐tert‐butylphenyl) diphenyl phosphate (ABS of 0.1), B4tBPPP: (2,4‐ditert‐butylphenyl) diphenyl phosphate (ABS of 0.1). Numerical values are listed in Supporting Information 6 Table S8

Material content analysis performed by independent third‐party laboratories showed that no FRs were detected in the PUFs except for that used in the OPFR chairs. The presence of brominated FRs was scanned for but found none above the LOQ hence the values are not reported here. FR emission measurements inside the exposure chamber was focused on the four identified FRs from the OPFR chair, TPHP, 4tBPDPP, B4tBPPP, and T4tBPP, and these were found in the air, settled dust, and simulated dermal transfer samples (Figure 6). ADDs of FRs for adults, toddlers, and infants were calculated for the four simulated human exposure pathways including particle inhalation, and ultrafine particulates/volatile inhalation, ingestion, and dermal contact exposure (Figure 7 and in Supporting Information 6). The higher FR values obtained from the duplicate test samples were used to obtain the worst‐case exposure levels. This exposure data was limited, but it provides an exploratory evaluation of exposure potentials by different exposure routes as presented below.

#### 3.2.1 Inhalation

TPHP was detected in both volatile and airborne particle phases, and the predicted concentration in a living room with an average ventilation rate (0.45/h) was 0.11 ng/m^3^, much lower than one‐tenth TLV guideline of 300 μg/m^3^.^33^ Placing the OPFR chair in the bedroom would increase the TPHP concentration by a factor of 7 (with average ventilation) to 14 (low ventilation for high performance homes) compared to the concentration in the living room. TPHP was the only FR detected in the volatile phase. In the particle phase, 4tBPDPP was also detected at a trace level (0.026 ng/m^3^).

For the scenario of one chair in a living room with an average ventilation, inhalation was predicted to be the route resulting in the least FR exposure, accounting for less than 1% of total ADD values (summation of all exposure routes and FRs measured) for all ages (Figure 7). Toddlers had the highest predicted inhalation ADDs with 0.015 ng/kg/day of TPHP and 0.0003 ng/kg/day of 4tBPDPP. Infants' inhalation ADDs are at 96.4% and adults' inhalation ADDs are at 37.3% of toddlers' value.

#### 3.2.2 Settled dust

TPHP and 4tBPDPP were consistently detected in settled dust from the OPFR chairs but at or below LOQ, with an average TPHP level of 15.9 ng/m^2^ and 4tBPDPP level of 0.74 ng/m^2^. Ingestion exposure was predicted to be the second most significant exposure route for TPHP, and the primary exposure route for 4tBPDPP in children (Figure 7). FR ADD via ingestion was higher for children, especially infants, due to their frequent hand to mouth contact from touching settled dust. Infants have the highest ingestion ADD with 18.7 ng/kg/day (23.5% of total ADD) for TPHP and 1.02 ng/kg/day (80.8%) for 4tBPDPP. Ingestion ADDs for toddlers were 10.6 ng/kg/day (19.1% of total ADD) for TPHP and 0.58 ng/kg/day (76.4%) for 4tBPDPP. Ingestion ADD for adults accounted for less than 3% of total ADD for both TPHP and 4tBPDPP. Infant's ingestion ADDs were up to 3060 times higher than adult's ADDs due to higher absolute ingestion and lower bodyweights.

#### 3.2.3 Dermal exposure

Three out of four identified FRs in the OFPR chair were measured in dermal transfer samples, with the highest levels of 342 ng/m^2^ (below LOQ) for TPHP, 112 ng/m^2^ for 4tBPDPP and 37.5 ng/m^2^ for B4tBPPP (Figure 6). B4tBPPP was only detected in dermal exposure samples.

Dermal contact exposure was estimated to be the most significant exposure route for adults and children for TPHP. Infants have the highest dermal ADD of TPHP at 60.6 ng/kg/day (76.5% of total ADD), followed by toddlers at 45.1 ng/kg/day (80.9%). Dermal ADDs were three orders of magnitude higher than ingestion or inhalation for adults, males at 34.6 ng/kg/day and females at 33.6 ng/kg/day, making dermal transfer the most significant FR exposure. The dermal contact TPHP ADD for females was 1.0 ng/kg/day lower than males' due to the assumption that an average female is smaller than an average male therefore having a smaller contact surface area with a chair. Infants have the highest dermal ADD for 4tBPDPP as well at 0.24 ng/kg/day, though this value is two orders of magnitude smaller than that for TPHP. All B4tBPPP exposure was predicted to be from dermal contact since B4tBPPP was only detected in dermal contact samples. B4tBPPP ADDs were an order of magnitude smaller than 4tBPDPP ADDs and three orders of magnitude smaller than TPHP ADDs.

#### 3.2.4 Overall FR exposure

The total mass of FRs in each test sample was estimated using PUF's density, volume, and FR weight percentages. An OPFR chair was estimated to contain 23.0 g of TPHP, 44.8 g of 4tBPDPP, 8.6 g of B4tBPPP, and 0.47 g of T4tBPP totaling 76.9 g of FRs in one OPFR chair. When the measured airborne OPFR concentrations were converted to ERs assuming FRs were released steadily over the sampling period, one OPFR chair was predicted to release up to 9.67 ng/h of TPHP and 0.16 ng/h of 4tBPDPP. This estimation equates to a small fraction (0.04 ppb) of total FRs by weight being released in ambient air every hour. Assuming that all larger‐sized settling particles/dust fell inside the chamber at a constant rate within the 24 h sampling period, the settled dust ERs were in the same range as airborne ERs, leading to 0.05 ppb of total FRs by weight being released and settled on the floor per hour.

TPHP was measured as the highest level of FR available for human exposure, followed by 4tBPDPP and B4tBPPP. The predicted TPHP ADDs from the OPFR chair were 34.6, 55.8, and 79.3 ng/kg/day for adults, toddlers, and infants, respectively. 4tBPDPP exposure was the second highest with ADDs of 0.141, 0.761, and 1.26 ng/kg/day for adults, toddlers, and infants, respectively. B4tBPPP ADDs via dermal contact were 0.032, 0.042, and 0.056 ng/kg/day for adults, toddlers, and infants. The summation of all the OPFR ADDs from a chair, 34.7, 56.6, 79.4 ng/kg/day for adults, toddlers, and infants, respectively, account for about a tenth of the estimated daily intake of OPFRs in various US indoor environments presented by Kim et al.^34^

The exposure modeling showed that young children, the most susceptible population, likely receive the highest FR exposure. The total ADD was higher for infants and toddlers than for adults; total ADDs from the OPFR chair were 2.32 and 1.63 times higher for infants and toddlers respectively. Infants had the largest total ADD due to them having the largest dermal and ingestion ADDs.

## 4 DISCUSSION

The research methodology on measuring and assessing VOC and FR emissions and exposures from furniture was presented. The methodology entails test product management, product exposure during simulated use, sample collection and measurement for ingestion, inhalation, and dermal contact exposure potentials, and exposure modeling for VOCs and FRs. This included the use of an environmentally controlled chamber for product testing and extensive analytical procedures for the collection and analysis of air, dust, and dermal transfer samples. These techniques provide consistent and defined processes for measuring human exposure to VOCs, FRs, and other potential contaminants from the use of consumer products like furniture.

Results demonstrated that FR exposure potentials can be present with use of chairs manufactured with a FR added to the PUF for flammability management, and this exposure can occur by inhalation, ingestion, and dermal contact. The studied reactive FR chemically bound to the PUF did not demonstrate any known FR emission/exposure.

Indoor air concentrations of the sum of OPFRs (that included TPHP as one of the chemicals detected) have been measured throughout the world, as low as 5.2 ng/m^3^ and as high as 217 ng/m^3^.^35^, ^36^ Indoor air concentrations of TPHP specifically range between 0.5 ng/m^3^ in a private home in Napal^35^ to a maximum of 1.08 ng/m^3^ (mean of 0.12 ng/m^3^ in various indoor environments, eg, homes, cars, stores) in the US.^34^ Despite the unknown parameters such as room volume and ventilation of each studied rooms from other literature, the predicted concentration in a living room with one studied lounge chair (0.11 ng/m^3^) is comparable since it is 92% of the mean TPHP concentration from indoor environments in the US (0.12 ng/m^3^), 10% of the maximum TPHP concentration in the US (1.08 ng/m^3^), and 22% of the measured residential concentration in Nepal (0.5 ng/m^3^). The studied chair should make up a fraction of the total measured TPHP concentrations since most likely there are other sources of TPHP in the studied room.

During simulated chair use, VOC levels were low and associated with the chair construction materials. The FR technologies applied did not contribute to the VOC emissions. Based on presented exposure modeling, VOCs exposure is primarily dependent on the number of chairs and ventilation rates in the environment. Although measured levels were below commonly accepted indoor air quality criteria, their levels could also be influenced by materials and environmental conditions. Formaldehyde and acetaldehyde were the prevalent VOCs of concern. FR exposure modeling and assessment indicated that the primary human health concern is settled dust/dermal contact exposure, with children having greater vulnerability due to their hand to mouth transfer. The use of a barrier textile or chemically bound FRs in PUF were shown to be available technologies to minimize VOC and FR chemical exposures.

Applied FR technologies should be free from potentially toxic chemicals, but they should also meet their objective which is fire prevention. Harris et al.^19^ presents the coinciding results from a series of smoldering and open fire experiments conducted on the four types of chairs studied. In summary, the only FR technology that suppressed a match‐equivalent open flame placed on top of the chair from igniting completely was the barrier textile. The fire never burned through the woven‐fiberglass barrier textile to the PUF or filling, resulting in lower heat release rate and asphyxiant gas concentrations compared to NFR, RFR, and OPFR chairs that burned to their frames within 15 min. No significant flammability differences were observed amongst NFR, RFR, and OPFR chairs. All chairs did not pass the smolder resistance test despite the effectiveness of the barrier textile for an open flame test.

Technologies do exist where upholstered furniture can be protected from fire and pollutant exposure, as demonstrated by the barrier textile selected for this case study. The study also provided methods and data to evaluate human risks associated with FRs and other potential chemical exposures, which is relevant to protecting the health and safety of consumers and fire safety professionals.

## CONFLICT OF INTEREST

The authors declare no conflict of interest.

## AUTHOR CONTRIBUTION

Aika Davis: involved in investigation, methodology, data curation, formal analysis, and writing—original draft preparation. P. Barry Ryan: involved in supervision, conceptualization, methodology, data curation, and writing—review & editing. Jordan Cohen: involved in investigation and methodology. Debra Harris: involved in conceptualization, project administration, resources, and writing—review & editing. Marilyn Black: involved in conceptualization, project administration, methodology, data curation, supervision, and writing—review & editing.

## Supporting information

Supplementary Material
