# Supplementary material for: Chemical exposures from upholstered furniture with various flame retardant technologies
Source: Indoor Air. 2021 Feb 24;31(5):1473–83. doi: 10.1111/ina.12805 (PMC8451937; doi:10.1111/ina.12805)
Supplement: Supplementary file 1 — Supplementary Material [file INA-31-1473-s001.docx]

# Supplemental Information

“Chemical Exposures from Upholstered Furniture with Various Flame Retardant Technologies”

Aika Davis, P. Barry Ryan, Jordan A. Cohen, Debra Harris, Marilyn Black

## SI 1: Chair descriptions

#### NFR

The chairs all consisted of a polyurethane foam (PUF) seat cushion core wrapped with fiber filling enclosed in ticking textile then cover fabric. The back cushion was constructed similarly except a polyester loose filling was used instead of PUF for the filling. Armrests also used PUF with the four FR technologies placed in between the wooden frames and the cover fabric. The NFR chair represented the product as currently manufactured and sold by the furniture company.

#### OPFR

The PUF for the chair containing OPFR was prepared and provided by the furniture foam supplier using their standard formulation and preparation processes. The FR was selected by the foam supplier to be representative of a currently used FR by the furniture industry and was added to the foam at levels consistent with current standard practice. The specific FRs used in this study were not initially known, therefore, the PUFs were independently identified through chemical analysis by sonicating the PUF samples and analyzing the extracts by gas chromatography mass spectrometry (GC/MS) following methods in Stapleton et al.^1^ The OPFR PUF sample was identified to be a mixture of triphenyl phosphate (TPHP) and *tert*-butylated triarylphosphate (TBPP) isomers, also known as TBPP mix. The OPFR foam was found to contain 2.9% by weight of the TBPP mix, which is in agreement that applied total FR concentration is generally greater than 1%.^2^

#### RFR

The RFR chair represented a novel chemical FR technology that chemically bonds to the PUF during the polymerization process. This FR was proprietary, and the formulation was not provided. An independent chemical analysis of this PUF did not show any detection of a series of known halogen and non-halogen FRs. Based on the limited information received from the foam supplier, this FR was expected to be similar to the one that received New Chemicals Program P2 Recognition Project Award in 2008^3^ which eliminates unwanted FR emissions by binding the FR within the polymeric PUF structure.

#### BNFR

The BNFR’s woven fiberglass textile barrier is currently commercially available. The fiberglass textile barrier was also applied on the back cushion and the armrests underneath the cover fabric.

## SI 2: Exposure Testing Details

### Exposure Chamber

The chamber was validated for mixing and air exchange rate using a tracer gas method.^26^ Air supplied to the chamber was continuously monitored. Construction, operation, and validation processes followed standard guidelines from ISO/IEC 28360,^5^ ISO 16000-9,^6^ UL 2821,^7^ ECMA-328,^8^ and ASTM D6670.^9^

### Pneumatic sitting device

An automated pneumatic machine called Robiesitz™ was custom made for this study to simulate sitting activity during personal use of the chair. Robiesitz™ provided a 3.6 cm free-fall onto the chair cushion with a 56.7 kg weight. This mimics an average US male’s upper body sinking into the chair from a standing position. The fall height, weight, weight diameter, and other specific parameters followed the guidance in the BIFMA X5.4 Seat Durability Test.^10^ Robiesitz™ operated at one sitting per minute; with the weight in air for 30 seconds and then a drop and rest period on the chair for 30 seconds. The apparatus was constructed with stainless steel and metal to avoid chemical contamination and avoid it acting as a sink or source of target compounds.

### VOC and aldehyde analysis

using methodologies adapted from EPA TO-17,^11^ ISO 16000-6,^12^ and ASTM D6196,^13^

Each VOC was specifically identified using mass spectral databases and quantitated using multipoint calibration standards to 80+ chemicals commonly found in indoor air, if available. Mass spectral characteristics of more than 75,000 compounds available from the National Institute of Standards and Technology (NIST), the EPA, and the National Institutes of Health (NIH) were used along with a dedicated laboratory database of indoor air VOCs. TVOC measurements were made by adding all individual VOC responses obtained by the GC/MS and converting the total mass to a toluene equivalent.

Emissions of low molecular weight aldehydes up to hexanal were collected onto solid sorbent cartridges treated with 2, 4-dinitrophenylhydrazine (DNPH) was analyzed by high-performance liquid chromatography (HPLC) following EPA TO-11A,^14^ ISO 16000-3,^15^ and ASTM D5197.^16^

## SI 3: Extraction and Analysis of Samples for FR Analysis


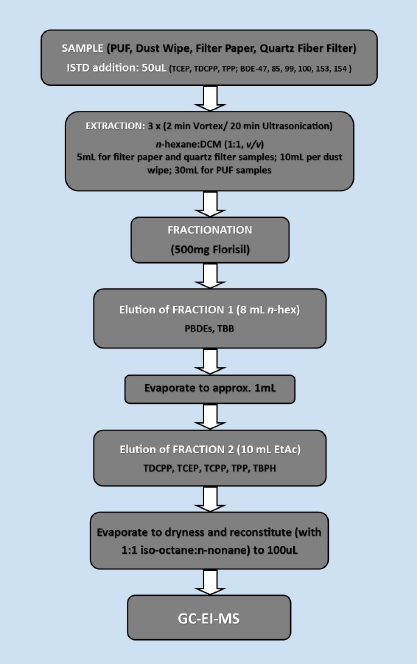


SI Figure 1: Schematic representation of the analytical procedure

Polyurethane foam (PUF) air sampling cartridges, quartz fiber filters, dust samples collected via wipe method, and samples collected using filter paper method are extracted separately. SI Figure 1 outlines the analytical procedure for FR analysis.

### Target analytes

Target brominated FR analytes:

1. 2,2',4,4'-tetraBDE (BDE-47) (13C12, 99%) (CIL part no EO-4982)
2. 2,2',4,4',6-pentaBDE (BDE-100) (13C12, 99%) (CIL part no EO-4993)
3. 2,2',4,4',5-pentaBDE (BDE-99) (13C12, 99%) (CIL part no EO-4983)
4. 2,2',4,4',5,6'-hexaBDE (BDE-154) (13C12, 99%) (CIL part no EO-5161)
5. 2,2',4,4',5,5'-hexaBDE (BDE-153) (13C12, 99%) (CIL part no EO-4984)

Target organophosphate FR analytes:

1. Tris(2-chloroethyl) phosphate D12 (TCEP),
2. Tris(1,3-dichloro-2-propyl) phosphate D15 (TDCPP)
3. Triphenyl phosphate D15 (TPHP)
4. (4-tert-butylphenyl) diphenyl phosphate (4tBPDPP)
5. (2,4-di-tert-butylphenyl) diphenyl phosphate (B4tBPPP)
6. tris(4-tert-butylphenyl) phosphate (T4tBPP)

### Sample extraction

Extraction procedure is as follows.

1. Selected the size of the extraction vessel and the volume of 1:1 Dichloromethane (DCM) and *n*-hexane to be used based on the sampling medium.
   1. Typical vessel size and solvent volume used for a low-volume PUF plug is a 50 mL conical vial with 30 mL solvent,
   2. for a quartz fiber filter, a 15 mL centrifuge tube with 5 mL solvent,
   3. for a filter paper sample, a 15 mL centrifuge tube with 5 mL solvent,
   4. and for a dust wipe sample, a 15 mL centrifuge tube with 10 mL solvent.
2. Placed the sample in the appropriate tube, and the appropriate volume of 1:1 DCM and n-hexane.
   1. 5 mL for filter paper and quartz filter
   2. 10mL for dust wipe
   3. 30 mL for PUF cartridge

For QA/QC, added 50μL of each calibration standard for both OPFRs and PBDEs

1. Capped the test tube and vortexed the sample for 2 min.

### Analytical Instrument

The GC/MS selected-ion-monitoring was set up to analyze for primary and secondary monitored ions for the different class target analytes. The GC oven temperature reached to 325 °C max at +45 °C /min, with +5 °C /min between 250 °C to 275 °C. Mass spectrometer scanned from 35 to 500 m/z at 1 s per scan.

Calibration standard was prepared at ten levels ranging from 0.2 to 2000ng/mL for both brominated and organophosphate FRs. Internal standard injected and the mass spectrum of each chromatographic peak was compared to the reference mass spectra of the target analytes for positive identification. Each sample data was analyzed for target analytes by criteria including retention times, mass spectra, signal to noise (S/N) ratio, and unique ion profiles. Any samples with a S/N of <3.0 were not quantified.

### QA/QC

At least 10% reagent blanks, procedural blanks, laboratory blanks, field blanks, and travel blanks were analyzed. Duplicates were collected for all samples collected.

Internal standard spiking solution of polybrominated diphenyl ethers is prepared to contain 150 pg/μL of BDE-47, 85, 99, 100, 153, 154 in *n-*nonane. An internal standard spiking solution of organophosphate flame retardants was prepared to contain 144pg/μL TDCPP, TCEP, TPHP, 4tBPDPP, B4tBPPP, and T4tBPP in acetonitrile.

Other QC standards are prepared separately at three levels, low at 10ng/mL, medium at 50ng/mL, and high varying concentrations of different compounds depending on the sample range. This is because of high background concentrations of these compounds in the extraction solvent and sampling media.

QC samples should be interspersed evenly throughout the run, between sample extracts, and each QC should be injected twice throughout the run to check for accuracy. Solvent blanks can be inserted where necessary to minimize carry-over from samples with known high concentrations.

## SI 4: Exposure modeling parameters

### Inhalation

The amount of pollutant (VOC or FR) inhaled over the duration of a daily exposure was calculated using a predicted airborne concentration expected for a residential environment. An emission factor of inhalable FR (${EF}_{FR, I})$ from the chair, as obtained from the exposure chamber measurement was first calculated following Equation 1,

${{EF}_{FR, I}=C}_{FR, I}\times\frac{V_{chamber}\times N_{chamber}}{A_{chamber}}$ (1)

where $C_{FR, I}$ is the FR airborne sample concentrations from PUF cartridge and quartz filter measured at steady state, $V_{chamber}$ is the volume of a chamber, $N_{chamber}$ is the air exchange rate inside the chamber, $A_{chamber}$ is the product loading inside the chamber or in this case one unit of chair.

${EF}_{FR, I}$ was used to predict air concentration for a defined residential environment ($C_{model,FR, I}$),

$C_{model,FR, I}={EF}_{FR, I} \times\frac{A_{model}}{V_{model}\times N_{model}}$ (2)

where $A_{model}$ is the test product loading in a model room, $V_{model}$ is the volume of the model room, $N_{model}$ is the air exchange/ventilation rate in a model room. For VOCs, predicted air concentrations are modeled to various room settings and compared to indoor air regulatory requirements and guidelines. For predicted ambient FR concentrations, Equation 3 was used to calculate an ADD of FR via inhalation by *i*, an adult, toddler, or infant ${(ADD}_{FR,I,i}$),

${ADD}_{FR, I, i}=C_{model,FR, I} \times Q_{inhalation, i}\times\frac{t_{exposure,i}}{24 hours}\times\frac{1}{{BW}_{i}}$ (3)

$Q_{inhalation, i}$ is the inhalation rate, $t_{exposure,i}$ is the time of exposure for a person, ${BW}_{i}$ is the average body weight of a person.

The default loading for exposure modeling was the use of one chair in an open floor residential setting combining living and dining areas. The single-family residential model used for exposure prediction was obtained from [ANSI/CAN/UL 2904](https://ulchemicalsafety.org/wp-content/uploads/2019/02/3DPrintStandard_Brief.pdf).^17^ The residential model in this standard is based on data from Appendix B of the California Department of Public Health Standard Method (CDPH SM)^18^ and the 2008 US Department of Energy (DOE) Buildings Energy Data Book.^19^ Age-specific daily inhalation rate and average body weight were obtained from the EPA Exposure Factor Handbook.^20^

#### Inhalation exposure modeling parameters

SI Table 1: Adult, Toddler, and Infant Factors for Inhalation Exposure

| Inhalation Exposure Factors | Adult | Toddler  (1-2 years) | Infant  (3-6 months) | Unit | Source |
| --- | --- | --- | --- | --- | --- |
| *A _model_* | 1 | 1 | 1 | Chair |  |
| *V_model_* (Residential living/dining) | 201 | 201 | 201 | m^3^ | UL 2904 |
| *V_model_* (bedroom) | 28.2 | 28.2 | 28.2 | m^3^ | UL 2904 |
| *N_model, avg_* | 0.45 | 0.45 | 0.45 | hr^-1^ | ASHRAE 62.1^21^ |
| *N_model, low ACH_* | 0.23 | 0.23 | 0.23 | hr^-1^ | UL 2904, ASHRAE 62.2^2^ |
| *Q_inhalation_*  (inhalation rate) | 16.0 | 8.0 | 4.1 | m^3^/day | EFH T6-1 |
| $t$*_exposure_* | 6.9 | 4.9 | 6.0 | hr | EFH T16-1 and 16-15 and 16 |
| *BW* | 80 | 11.4 | 7.4 | kg | EFH T8-1 |

EFH: EPA Exposure Factors Handbook^20^
UL 2904: ANSI/CAN/UL 2904^17^

### Ingestion

Saliva mediated oral exposure quantity was determined as the amount of FR ingested during daily exposure, directly and indirectly. A combination of dust to hand to mouth contact (Equation 4), chair surface/fabric to hand to mouth contact (Equation 5), and chair surface to mouth contact (Equation 6), constitute the total amount of FR ingested (Equation 7). The mathematical models for ingestion exposure were based on Keil et al.^23^ and Babich.^24^

The ADD from dust to hand to mouth contact (${ADD}_{FR,O1,i}$, Equation 4) was calculated from the settled dust sample concentration of a FR ($C_{FR, O}$ ) measured in the exposure chamber study multiplied by the fraction of dust transferred from ground to hands ($f_{gh}),$the total surface area of one side of both hands (${SA}_{h,i}),$the rate of hand to mouth contacts ($r_{contacts,i})$, $t_{exposure,i}$, scaled by ${BW}_{i}$.

${ADD}_{FR,O1,i}=C_{FR,O}\times f_{gh}\times{SA}_{h,i}{\times r}_{contacts,i}\times t_{exposure,i}\times\frac{1}{{BW}_{i}}$ (4)

The ADD from the chair surface to hand to mouth contact (${ADD}_{FR,O2,i}$, Equation 5) was calculated by taking the measured simulated dermal filter patch sample concentration ($C_{FR,D})$, normalizing this value for the exposure duration ($t_{exposure,i}$/ $t_{sampling}$). Only a fraction of this normalized dermal concentration was transferred from the chair fabric to hand and then hand to mouth using the following parameters: the fraction transferred from fabric to hand ($f_{fh}$), ${SA}_{h, i}$, and hand to mouth transfer factor ($F_{hm}$). This value was scaled by ${BW}_{i}$.

${ADD}_{FR,O2,i}=C_{FR,D}\times\frac{t_{exposure,i}}{t_{sampling}}\times\left( f_{fh}\times{SA}_{h, i}\times F_{hm} \right)\times\frac{1}{{BW}_{i}}$ (5)

Since the chair surface to hand to mouth exposure model was directly from Babich,^24^ the parameters used in the reference were used. Due to the lack of data available for the FRs of interest, $f_{fh}$ was the moist fraction transferred for tris(1,3-dichloro-2-propyl)phosphate (TDCPP) in Babich,^24^ assuming that other FRs in this study have a similar transfer factor as TDCPP.

The ADD from chair surface directly to mouth (${ADD}_{FR,O3,i}$, Equation 6) was calculated by taking $C_{FR,D}$, normalizing this value for the exposure duration ($t_{exposure,i}$/ $t_{sampling}$) and taking into account for the directly mouthed surface area (${SA}_{m, i}$) and the fabric to mouth transfer factor ($F_{fm}$). This value was scaled by ${BW}_{i}$ to get to the ADD from chair surface to mouth exposure.

${ADD}_{FR,O3,i}=C_{FR,D}\times\frac{t_{exposure,i}}{t_{sampling}}\times\left( {SA}_{m, i}\times F_{fm} \right)\times\frac{1}{{BW}_{i}}$ (6)

The total ADD from ingestion exposure (${ADD}_{FR,O,i}$) is modeled as the sum of all the indirect (dust to hand to mouth contact and chair surface/fabric to hand to mouth contact) and direct (chair surface to mouth) ingestion exposures calculated above (Equation 7).

${ADD}_{FR,O,i}={ADD}_{FR,O1,i}+{ADD}_{FR,O2,i}+{ADD}_{FR,O3,i}$ (7)

#### Ingestion exposure modeling parameters

The factors used for dust to hand to mouth exposure are listed in SI Table 2.

SI Table 2: Adult, Toddler, and Infant Factors for Dust to Hand-to-Mouth Oral Exposure

| Oral Exposure Factors | Adult | Toddler  (1-2 years) | Infant  (3-6 months) | Unit | Source |
| --- | --- | --- | --- | --- | --- |
| $f_{gh}$ | 0.05 | 0.05 | 0.05 | - | Babich (2006) T8 |
| *SA _h_* | 0.049 | 0.015 | 0.010 | m^2^ | EFH T7-2 |
| *r_contacts_* (hand to mouth contacts) | 0 | 20 | 28 | Contacts/hr | EFH T4-1 |
| $t$ *_exposure_* | 6.9 | 4.9 | 6.0 | hr | EFH T16-1 and 16-15 and 16-16 |
| *BW* | 80 | 11.4 | 7.4 | kg | EFH T8-1 |

EFH: EPA Exposure Factors Handbook^20^
Babich (2006)^24^

The factors used for chair surface to hand-to-mouth exposure are listed in SI Table 3.

SI Table 3: Adult, Toddler, and Infant Factors for Chair Surface to Hand-to-Mouth Oral Exposure Modeling

| Oral Exposure Factors | Adult | Toddler (1-2 years) | Infant  (3-6 months) | Unit | Source |
| --- | --- | --- | --- | --- | --- |
| $t$ *_exposure_* | 6.9 | 4.9 | 6.0 | hr | EFH T16-1 and 16-15 and 16-16 |
| $t$ *_sampling_* | 6.0 | 6.0 | 6.0 | hr |  |
| $f_{fh}$ | 0.06 | 0.06 | 0.06 | - | Babich (2006) T8 |
| *SA_h_* | 0.049 | 0.015 | 0.010 | m^2^ | EFH T7-2 |
| $F_{hm}$ | 0.43 | 0.43 | 0.43 | /day | Babich (2006) T9 and Hatlelid (2005) |
| *BW* | 80 | 11.4 | 7.4 | kg | EFH T8-1 |

EFH: EPA Exposure Factors Handbook^20^
Babich (2006)^24^
Hatlelid (2005)^25^

The factors used for direct chair surface-to-mouth exposure are listed in SI Table 4.

SI Table 4: The Adult, Toddler, and Infant Specific Parameters Used for Chair Surface to Mouth Oral Exposure Modeling

| Oral exposure factors | Adult | Toddler (1-2 years) | Infant (3-6 months) | Unit | Source |
| --- | --- | --- | --- | --- | --- |
| $t$ *_exposure_* | 6.9 | 4.9 | 6.0 | hr | EFH T16-1 and 16-15 and 16-16 |
| $t$ *_sampling_* | 6.0 | 6.0 | 6.0 | hr |  |
| SA_m_ | 0 | 0.001 | 0.001 | m^2^ | Babich (2006) T 9 |
| $F_{fm}$ | 0.43 | 0.43 | 0.43 | /day | Babich (2006) T 9 and Hatlelid (2005) |
| BW | 80 | 11.4 | 7.4 | kg | EFH T8-1 |

EFH: EPA Exposure Factors Handbook^20^
Babich (2006)^24^
Hatlelid (2005)^25^

### Dermal Contact

Dermal contact was assumed to be sweat-mediated through exposed skin directly in contact with the chair surface. Sweat mediated dermal exposure prediction was based on Keil et al.^23^ and Thomas et al.^26^ $C_{FR,D}$ was normalized for the exposure duration ($t_{exposure,i}$/ $t_{sampling}$) and defined by the area of skin in contact with the chair (${SA}_{contact,i}$) while in use, and the fraction absorbed by the skin (*ABS*). This amount was scaled by ${BW}_{i}$ to calculate the ADD via dermal exposure (Equation 8).

${ADD}_{FR,D,i}=C_{FR,D}\times\frac{t_{exposure,i}}{t_{sampling}}\times{SA}_{contact,i}\times ABS\times\frac{1}{{BW}_{i}}$ (8)

The age-specific surface area in contact with a chair was calculated as one-third area of each body part listed: trunk, legs, arms, and hands. One-third was assumed to account for the curvature of limbs, and the surface area in contact would scale with the fractional assumption. This is a conservative estimate since it assumes a person being shirtless; therefore, a discussion with the average daily dose decreasing linearly with a fraction exposed would be warranted. The largest human dermal absorption factor found by Pawar et al.,^27^ ABS of 0.18, was used for TPHP. For the other FRs where specific ABS factors were not obtainable, ABS of 0.1 was applied since it is the recommended value for semi-volatile organic compounds by the EPA.^28^ ABS of 1.0 was also applied as a worst case scenario.^23^

#### Dermal exposure modeling parameters

The factors used for dermal exposure are listed in SI Table 5.

SI Table 5: Adult Male and Female, Toddler, and Infant Factors for Dermal Exposure

| Dermal Exposure Factors | Adult male | Adult female | Toddler | Infant | Unit | Source |
| --- | --- | --- | --- | --- | --- | --- |
| $t$ _exposure_ | 6.9 | 6.9 | 4.9 | 6.0 | hr | EFH T16-1 and 16-15 and 16-16 |
| $t$ _sampling_ | 6.0 | 6.0 | 6.0 | 6.0 | hr |  |
| SA_contact_ | 0.64 | 0.53 | 0.14 | 0.10 | m^2^ | EFH T 7-2 |
| ABS_TPHP_ | .18 | .18 | .18 | .18 | - | Pawar (2017) |
| ABS_all other FR_ | .1 | .1 | .1 | .1 | - | EPA (2007) |
| ABS for worst case | 1 | 1 | 1 | 1 | - | Keil (2009) |
| BW | 89 | 76 | 11.4 | 7.4 | kg | EFH T8-1, 4(male), 5(female) |

EFH: EPA Exposure Factors Handbook^20^
Pawar (2017): Pawar doctoral thesis^27^

EPA (2007): Dermal Exposure Assessment^28^
Keil (2009): Keil et al.^23^

## SI 5: Complete list of VOC emission rates and predicted exposure concentrations

SI Table 6: List of Individual VOCs and their Average Emission Rate (μg/hr/chair).

| CAS No | Chemical | No FR (control) | OPFR | Reactive FR  (RFR) | Barrier textile  (BNFR) |
| --- | --- | --- | --- | --- | --- |
| Total | TVOC | 596 | 1090 | 310 | 794 |
| 66-25-1 | Hexanal | 322 | 563 | 116 | 428 |
| 79-09-4 | Propanoic acid | 76.1 | 194 | 145 | 191 |
| 71-36-3 | 1-Butanol | 170 | 24.9 | 22.2 | 230 |
| 108-32-7 | Propylene Carbonate | NQ | 284 | NQ | NQ |
| 110-62-3 | Pentanal | 57.4 | 114 | 18.0 | 93.0 |
| 123-38-6 | Propanal | 43.7 | 117 | NQ | 68.7 |
| 142-62-1 | Hexanoic acid | 32.3 | 33.9 | 20.6 | 69.3 |
| 108-95-2 | Phenol | NQ | 179 | NQ | NQ |
| 75-07-0 | Acetaldehyde | 62.9 | 50.4 | 17.7 | 66.3 |
| 111-76-2 | Ethanol, 2-butoxy | 45.5 | 44.4 | 28.5 | 68.7 |
| 149-57-5 | 2-Ethylhexanoic acid | 52.0 | NQ | 23.6 | 60.3 |
| 98-01-1 | Furfural | NQ | 42.9 | 21.5 | 58.2 |
| 50-00-0 | Formaldehyde | 25.2 | 24.0 | 27.6 | 39.0 |
| 104-76-7 | 1-Hexanol, 2-ethyl | 22.0 | 12.9 | 18.3 | 55.5 |
| 71-41-0 | 1-Pentanol (N-Pentyl alcohol) | 33.7 | 37.5 | NQ | 42.0 |
| 116-09-6 | 2-Propanone, 1-hydroxy | NQ | 53.7 | 15.3 | NQ |
| 106-46-7 | Dichlorobenzene (1,4-) | NQ | NQ | NQ | NQ |
| 107-98-2 | Propylene glycol monomethyl ether (2-Propanol, 1-methoxy-) | NQ | 107.1 | NQ | NQ |
| 100-52-7 | Benzaldehyde | 19.5 | 21.6 | NQ | 32.1 |
| 107-92-6 | Butanoic acid | NQ | 33.3 | 20.1 | NQ |
| 1066-42-8 | Silanediol, dimethyl- | 20.4 | NQ | NQ | 19.2 |
| 124-19-6 | Nonyl aldehyde (Nonanal) | NQ | 12.0 | NQ | 12.9 |
| 5077-67-8 | 1-Hydroxy-2-butanone | NQ | 17.7 | NQ | 15.9 |
| 96-48-0 | Butyrolactone | NQ | NQ | NQ | 35.1 |
| 541-05-9 | Cyclotrisiloxane, hexamethyl | 15.5 | 12.3 | NQ | 19.8 |
| 96-37-7 | Cyclopentane, methyl | NQ | NQ | NQ | NQ |
| 13429-07-7 | 2-Propanol, 1-(2-methoxypropoxy)- | NQ | 13.5 | NQ | NQ |
| 107-52-8 | Hexasiloxane, tetradecamethyl | NQ | 19.5 | NQ | NQ |
| 556-67-2 | Cyclotetrasiloxane, octamethyl | 14.0 | NQ | NQ | 17.4 |
| 20324-33-8 | 2-Propanol, 1-[2-(2-methoxy-1-methylethoxy)-1-methylethoxy]- | NQ | 13.8 | NQ | NQ |
| 108-65-6 | 1-Methoxy-2-propyl acetate | NQ | 15.9 | NQ | 12.9 |
| 123-72-8 | Butanal | NQ | 13.2 | NQ | 12.9 |
| 629-50-5 | Tridecane | NQ | 24.3 | NQ | NQ |
| 6846-50-0 | TXIB | NQ | NQ | NQ | 21.3 |
| 629-59-4 | Tetradecane | NQ | 19.2 | NQ | NQ |
| 141-63-9 | Pentasiloxane, dodecamethyl | NQ | 18.9 | NQ | NQ |
| 1569-02-4 | 2-Propanol, 1-ethoxy | NQ | 18.0 | NQ | NQ |
| 68-12-2 | Formamide, N,N-dimethyl | NQ | NQ | NQ | NQ |
| 57-55-6 | Propylene glycol | NQ | 15.0 | NQ | NQ |
| 497-26-7 | 1,3-Dioxolane, 2-methyl | NQ | 13.2 | NQ | NQ |

NQ: not quantifiable since chamber concentrations used to calculate the emission rate were below LOQ of 2 μg/m^3^

SI Table 7: List of VOC criteria by Ausschuss zur gesundheitlichen Bewertung von Bauprodukten’s Lowest Concentration of Interest (AgBB LCI),^29^ the American Conference of Governmental Industrial Hygienists’ Threshold Limit Values (ACGIH TLV^®^),^30^ and the California Department of Public Health Standard Method (CDPH SM),^18^ maximum predicted living/dining room concentrations, maximum predicted bedroom concentrations, all in μg/m^3^.

| CAS No. | Chemical | Living room conc. | Bedroom conc. | AgBB  LCI | ACGIH  1/10 TLV | CDPH SM |
| --- | --- | --- | --- | --- | --- | --- |
| 000-00-0 | TVOC | 12.1 | 168 | NA | NA | NA |
| 66-25-1 | Hexanal | 6.22 | 86.8 | 900 | NA | NA |
| 79-09-4 | Propanoic acid | 3.34 | 46.6 | 310 | NA | NA |
| 71-36-3 | 1-Butanol | 2.54 | 35.5 | 3000 | 6063 | NA |
| 108-32-7 | Propylene Carbonate | 3.14 | 43.8 | 250 | NA | NA |
| 110-62-3 | Pentanal | 1.26 | 17.6 | 800 | NA | NA |
| 123-38-6 | Propanal | 1.29 | 18.0 | NA | NA | NA |
| 142-62-1 | Hexanoic acid | 0.803 | 11.2 | 490 | NA | NA |
| 108-95-2 | Phenol | 1.98 | 27.6 | 10 | 1925 | 100 |
| 75-07-0 | Acetaldehyde | 0.733 | 10.2 | 1200 | 4504 | 70 |
| 111-76-2 | Ethanol, 2-butoxy | 0.760 | 10.6 | 1100 | 9666 | NA |
| 149-57-5 | 2-Ethylhexanoic acid | 1.19 | 16.7 | 150 | NA | NA |
| 98-01-1 | Furfural (2-Furaldehyde) | 0.643 | 8.97 | 20 | 79 | NA |
| 50-00-0 | Formaldehyde | 0.431 | 6.01 | 100 | 12 | 9 |
| 104-76-7 | 1-Hexanol, 2-ethyl | 0.627 | 8.74 | 300 | NA | NA |
| 71-41-0 | 1-Pentanol | 0.464 | 6.48 | 730 | NA | NA |
| 116-09-6 | 2-Propanone, 1-hydroxy | 0.594 | 8.28 | 2400 | NA | NA |
| 106-46-7 | Dichlorobenzene (1,4-) | 0.464 | 6.48 | NA | 6012 | 400 |
| 107-98-2 | Propylene glycol monomethyl ether | 1.18 | 16.5 | 3700 | NA | 3500 |
| 100-52-7 | Benzaldehyde | 0.355 | 4.95 | 90 | NA | NA |
| 107-92-6 | Butanoic acid | 0.368 | 5.13 | 370 | NA | NA |
| 1066-42-8 | Silanediol, dimethyl- | 0.226 | 3.15 | NA | NA | NA |
| 124-19-6 | Nonyl aldehyde (Nonanal) | 0.163 | 2.27 | 900 | NA | NA |
| 5077-67-8 | 1-Hydroxy-2-butanone | 0.205 | 2.85 | NA | NA | NA |
| 96-48-0 | 2(3H)-Furanone, dihydro (Butyrolactone) | 0.388 | 5.41 | 2700 | NA | NA |
| 541-05-9 | Cyclotrisiloxane, hexamethyl | 0.219 | 3.05 | NA | NA | NA |
| 96-37-7 | Cyclopentane, methyl | 0.517 | 7.22 | NA | NA | NA |
| 13429-07-7 | 2-Propanol, 1-(2-methoxypropoxy)- | 0.169 | 2.36 | NA | NA | NA |
| 107-52-8 | Hexasiloxane, tetradecamethyl | 0.216 | 3.01 | NA | NA | NA |
| 556-67-2 | Cyclotetrasiloxane, octamethyl | 0.192 | 2.68 | 1200 | NA | NA |
| 20324-33-8 | 2-Propanol, 1-[2-(2-methoxy-1-methylethoxy)-1-methylethoxy]- | 0.168 | 2.34 | 2000 | NA | NA |
| 108-65-6 | 1-Methoxy-2-propyl acetate | 0.176 | 2.45 | 2700 | NA | NA |
| 123-72-8 | Butanal | 0.146 | 2.04 | 650 | NA | NA |
| 629-50-5 | Tridecane | 0.269 | 3.75 | NA | NA | NA |
| 6846-50-0 | TXIB | 0.235 | 3.28 | 450 | NA | NA |
| 629-59-4 | Tetradecane | 0.212 | 2.96 | NA | NA | NA |
| 141-63-9 | Pentasiloxane, dodecamethyl | 0.209 | 2.91 | NA | NA | NA |
| 1569-02-4 | 2-Propanol, 1-ethoxy | 0.199 | 2.78 | NA | NA | NA |
| 68-12-2 | Formamide, N,N-dimethyl | 0.182 | 2.54 | 15 | 1495 | 40 |
| 57-55-6 | Propylene glycol | 0.166 | 2.31 | 2500 | NA | NA |
| 497-26-7 | 1,3-Dioxolane, 2-methyl | 0.146 | 2.04 | NA | NA | NA |
| 103-09-3 | Acetic acid, 2-ethylhexyl ester | 0.143 | 1.99 | 350 | NA | NA |
| 128-37-0 | 2,6-Di-tert-butyl-4-methylphenol (BHT) | 0.139 | 1.94 | 100 | NA | NA |
| 541-02-6 | Cyclopentasiloxane, decamethyl | 0.133 | 1.85 | 1500 | NA | NA |

NA stands for not available.

Maximum predicted living/dining room concentrations were calculated using the maximum emission rates across all chairs with 201 m^3^ space with 0.45/hr air exchange rate, one chair per room.

Maximum predicted bedroom concentrations were calculated using the maximum emission rates across all chairs with 28.2 m^3^ space with 0.23/hr air exchange rate.

## SI 6: Predicted average daily doses (ADDs) in ng/kg/day from Figure 7

SI Table 8: Predicted average daily doses (ADDs) in ng/kg/day for male, female adults, toddler, and infant. ADDs are separated by gas phase and particle phase inhalation, ingestion, and simulated dermal exposure routes followed by the total/summation of all exposure routes for the three FRs detected during exposure chamber testing. TPhP: triphenyl phosphate, 4tBPDPP: (4-tert-butylphenyl) diphenyl phosphate, B4tBPPP: (2,4-ditert-butylphenyl) diphenyl phosphate.

|  | Male adult | | |  | Female adult | | |  | Toddler | | |  | | Infant | | | |
| --- | --- | --- | --- | --- | --- | --- | --- | --- | --- | --- | --- | --- | --- | --- | --- | --- | --- |
|  | TPHP | 4tBPDPP | B4tBPPP |  | TPHP | 4tBPDPP | B4tBPPP |  | TPHP | 4tBPDPP | B4tBPPP | |  | | TPHP | 4tBPDPP | B4tBPPP |
| Gas phase inhalation | 3.22E-3 |  |  |  | 3.22E-3 |  |  |  | 8.63E-3 |  |  | |  | | 8.32E-3 |  |  |
| Particle phase inhalation | 2.51E-3 | 9.20E-5 |  |  | 2.51E-3 | 9.20E-5 |  |  | 6.73E-3 | 2.47E-4 |  | |  | | 6.49E-3 | 2.38E-4 |  |
| Ingestion | 6.09E-3 | 3.09E-3 |  |  | 6.09E-3 | 3.09E-3 |  |  | 1.06E+1 | 5.81E-1 |  | |  | | 1.87E+1 | 1.02E+0 |  |
| Simulated dermal contact^†^ | 3.46E+1 | 1.38E-1 | 3.20E-2 |  | 3.36E+1 | 1.34E-1 | 3.10E-2 |  | 4.51E+1 | 1.80E-1 | 4.17E-2 | |  | | 6.06E+1 | 2.41E-1 | 5.61E-2 |
| Total | 3.46E+1 | 1.41E-1 | 3.20E-2 |  | 3.36E+1 | 1.37E-1 | 3.10E-2 |  | 5.58E+1 | 7.61E-1 | 4.17E-2 | |  | | 7.93E+1 | 1.26E+0 | 5.61E-2 |

^†^ The fraction absorbed by the skin (*ABS*) for TPHP is 0.18 and for the rest of FRs is 0.1.

# Reference

1. Stapleton HM, Sharma S, Getzinger G, et al. Novel and High Volume Use Flame Retardants in US Couches Reflective of the 2005 PentaBDE Phase Out. *Environ Sci Technol*. 2012;46:13432–13439.

2. Hammel SC, Hoffman K, Lorenzo AM, et al. Associations between flame retardant applications in furniture foam, house dust levels, and residents’ serum levels. *Environment international*. 2017;107:181–189.

3. US EPA. P2 Recognition Project.https://www.epa.gov/reviewing-new-chemicals-under-toxic-substances-control-act-tsca/p2-recognition-project. Published May 19, 2017. May 19, 2017. https://www.epa.gov/reviewing-new-chemicals-under-toxic-substances-control-act-tsca/p2-recognition-project.

4. ASTM. *ASTM E741-11: Standard Test Method for Determining Air Change in a Single Zone by Means of a Tracer Gas Dilution*. West Conshohocken, PA: ASTM International; 2011.

5. ISO. *ISO/IEC 28360 Information Technology — Office Equipment — Determination of Chemical Emission Rates from Electronic Equipment*. Geneva, Switzerland: International Organization for Standardization; 2018. 2018. https://www.iso.org/standard/74677.html.

6. ISO. ISO 16000-9 Indoor air – Part 9: Determination of the emission of volatile organic compounds from building products and furnishing – Emission test chamber method. *International Organization for Standardization*. 2007.

7. UL. *UL 2821 GREENGUARD Certification Program Method for Measuring and Evaluating Chemical Emissions From Building Materials, Finishes and Furnishings*. Northbrook, IL: Underwriters Laboratories Inc.; 2014.

8. ECMA. *ECMA-328 Standard 7th Edition, Determination of Chemical Emission Rates from Electronic Equipment*. Geneva, Switzerland: Ecma International; 2015.

9. ASTM. ASTM D6670-13 Standard Practice for Full-Scale Chamber Determination of Volatile Organic Emissions from Indoor Materials / Products. 2013.

10. BIFMA. *ANSI/BIFMA X5.4-2012 Lounge and Public Seating - Tests: American National Standard for Office Furnishings*. Washington DC, US: ANSI; 2012.

11. US EPA. Compendium of Methods for the Determination of Toxic Organic Compounds in Ambient Air Second Edition Compendium Method TO-17 Determination of Volatile Organic Compounds in Ambient Air Using Active Sampling Onto Sorbent Tubes. 1999.

12. ISO. ISO 16000-6 Indoor Air — Part 6: Determination of volatile organic compounds in indoor and test chamber air by active sampling on Tenax TA sorbent thermal desorption and gas chromatography using MS or MS-FID. 2011.

13. ASTM. ASTM D6196-15 Standard Practice for Choosing Sorbents , Sampling Parameters and Thermal Desorption Analytical Conditions for Monitoring Volatile Organic Chemicals in Air. 2015.

14. US EPA. Compendium of Methods for the Determination of Toxic Organic Compounds in Ambient Air Second Edition Compendium Method TO-11A Determination of Formaldehyde in Ambient Air Using Adsorbent Cartridge Followed by High Performance Liquid Chromatography ( HPLC). 1999.

15. ISO. ISO 16000-3 Indoor air — Part 3: Determination of formaldehyde and other carbonyl compounds in indoor air and test chamber air — Active sampling method. 2011.

16. ASTM. ASTM D5197-16 Standard Test Method for Determination of Formaldehyde and Other Carbonyl Compounds in Air (Active Sampler Methodology). 2016:1–16.

17. ANSI, Underwriters Laboratories Inc. *ANSI/CAN/UL 2904 Standard Method for Testing and Assessing Particle and Chemical Emissions from 3D Printers*. Washington DC, US: American National Standards Institute; 2019. January 31, 2019. https://www.shopulstandards.com/ProductDetail.aspx?UniqueKey=35397.

18. CDPH. *Standard Method for the Testing and Evaluation of Volatile Organic Chemical Emissions from Indoor Sources Using Environmental Chambers Version 1.2*. Sacramento, CA: California Department of Public Health; 2017.

19. US DOE. *2011 Buildings Energy Data Book*. US Department of Energy; 2012.

20. US EPA. *Exposure Factors Handbook 2011 Edition*. Cincinnati, OH: U.S. Environmental Protection Agency; 2011.

21. ANSI, ASHRAE. *ANSI/ASHRAE Standard 62.1-2016 Ventilation for Acceptable Indoor Air Quality*. Washington DC, US: American National Standards Institute; 2016.

22. ASHRAE. *ANSI/ASHRAE Standard 62.2-2016 Ventilation for Acceptable Indoor Air Quality in Low-Rise Residential Buildings*. Washington DC, US: American National Standards Institute; 2016.

23. Keil CB, Simmons CE, Anthony TR. *Mathematical Models for Estimating Occupational Exposure to Chemicals*. 2nd ed. Fairfax, VA: AIHA Press; 2009.

24. Babich MA. *CPSC Staff Preliminary Risk Assessment of Flame Retardant (FR) Chemicals in Upholstered Furniture Foam*. Bethesda, MD: US Consumer Product Safety Commission; 2006:85–212.

25. Hatlelid KM, Bittner PM, Midgett JD, Thomas TA, Saltzman LE. Exposure and Risk Assessment for Arsenic from Chromated Copper Arsenate (CCA)-Treated Wood Playground Equipment. *null*. 2005;2:215–241.

26. Thomas T, Brundage P. *Qualitative Assessment of Potential Health Effects From the Use of Fire Retardant Chemicals in Mattresses*. Bethesda, MD: US Consumer Product Safety Commission; 2006.

27. Pawar G. Exploring the utility of 3D-skin models to evaluate trans-dermal uptake of flame retardants from indoor dust and consumer products. June 2017. June 2017. https://etheses.bham.ac.uk/id/eprint/7690/.

28. US EPA. *Dermal Exposure Assessment: A Summary of EPA Approaches*. Cincinnati, OH: U.S. Environmental Protection Agency; 2007.

29. AgBB. Health-related Evaluation Procedure for Volatile Organic Compounds Emissions (VVOC, VOC and SVOC) from Building Products 1. 2015:1–26.

30. ACGIH. TLVs® and BEIs®: Threshold limit values for chemical substances and physical agents biological exposure indices. *Signature Publications*. 2018.
